# Resistance to Linezolid and Pretomanid in the Era of Modern Drug-Resistant Tuberculosis Treatment in South Africa: A Systematic Review and Meta-Analysis

**DOI:** 10.3390/antibiotics15060543

**Published:** 2026-05-28

**Authors:** Kabelo Gabriel Kaapu, Vukosi Treasure Makondo, Emilyn Costa Conceição, Ivy Rukasha

**Affiliations:** 1 Department of Pathology, School of Medicine, University of Limpopo, Sovenga 0727, Limpopo, South Africa; makondotreasure@gmail.com (V.T.M.); ivy.rukasha@ul.ac.za (I.R.); 2 Division of Molecular Biology and Human Genetics, South African Medical Research Council Centre for Tuberculosis Research, Faculty of Medicine and Health Sciences, Stellenbosch University, Cape Town 7505, Western Cape, South Africa; emilyncosta@sun.ac.za; 3 Department of Microbiology, Polokwane Laboratory, National Health Laboratory Service, Polokwane 0699, Limpopo, South Africa

**Keywords:** drug-resistant tuberculosis, linezolid, pretomanid, antimicrobial resistance, minimum inhibitory concentration, whole-genome sequencing, South Africa

## Abstract

Background: The success of modern drug-resistant tuberculosis (DR-TB) regimens increasingly depends on linezolid (LZD) and pretomanid (Pa), yet the emergence of resistance to these critical agents threatens to reverse recent treatment advances, with limited consolidated evidence available from high-burden settings such as South Africa. Objectives: To systematically review and meta-analyse South African data on LZD and Pa resistance, minimum inhibitory concentrations (MICs), resistance-associated mutations, and treatment outcomes. Eligibility Criteria: We included clinical trials, cohort studies, surveillance studies, and molecular investigations conducted in South Africa from 2013 onward that reported resistance prevalence, MIC data, genotypic mutations, or treatment outcomes related to LZD and/or Pa. Information Sources: PubMed, PubMed, Embase, Web of Science, and grey literature sources were searched from January 2013 to 31 December 2025 in accordance with PRISMA 2020 guidelines. Risk of Bias: Study quality was assessed using the Joanna Briggs Institute (JBI) cohort appraisal checklist. Included Studies: Seventeen studies representing provincial and national cohorts were included. Synthesis of Results: Random-effects meta-analysis was used to estimate pooled baseline resistance. Subgroup, sensitivity, and meta-regression analyses were performed. Results: Random-effects meta-analysis demonstrated a pooled baseline LZD resistance prevalence of 0.53% (95% CI: 0.01–1.83; I^2^ = 81.1%) in routine South African cohorts, while substantially higher resistance (33%) was observed in treatment-failure populations. Baseline LZD MICs were typically 0.125–1.0 µg/mL, while elevated MICs (up to 8.0 µg/mL) were associated with *rplC* and *rrl* mutations, particularly *rplC Cys154Arg*. No confirmed phenotypic Pa resistance was identified across included South African cohorts, despite the detection of resistance-associated mutations in genomic surveillance studies. MIC values remained within the range of 0.016–1.0 µg/mL. Mutations in *ddn*, *fbiA*, *fbiC*, and *fgd1* were reported in genomic studies. Treatment success rates ranged from 63.6% to 99% for LZD-containing regimens and approached 90% for Pa-based regimens. Limitations: Limited study numbers, heterogeneity in laboratory methods, and overrepresentation of certain provinces may affect generalizability. Conclusions: Baseline resistance to LZD and Pa in South Africa remains low, supporting continued programmatic use. Ongoing molecular surveillance is essential to detect resistance amplification and preserve regimen efficacy.

## 1. Introduction

Tuberculosis (TB), caused by *Mycobacterium tuberculosis* (MTB), remains one of the leading causes of death from a single infectious agent globally, surpassing HIV/AIDS. The World Health Organization estimates that approximately one-quarter of the global population is infected with MTB, underscoring the persistent burden of TB worldwide. Drug-resistant TB (DR-TB) further intensifies this public health crisis, with approximately 3–4% of new cases and more than 15% of previously treated cases classified as rifampicin-resistant or multidrug-resistant TB, accounting for nearly 400,000 cases annually. Despite advances in diagnosis and treatment, treatment success in many high-burden settings remains below 60%, highlighting the urgent need for shorter, safer, and more effective treatment regimens [1,2]. Historically, DR-TB treatment relied heavily on second-line injectable agents, including aminoglycosides such as kanamycin and amikacin as well as the cyclic peptide capreomycin, which were associated with prolonged treatment duration, painful administration, severe adverse effects, high rates of treatment interruption, and poor patient acceptability. These limitations contributed to suboptimal outcomes and reinforced the need for all-oral regimens with improved efficacy, tolerability, and feasibility in programmatic settings [3,4].

South Africa is globally recognized as a leader in the rapid adoption and programmatic implementation of novel treatment strategies for drug-resistant tuberculosis (DR-TB), driven by one of the highest burdens of rifampicin-resistant (RR-TB) and multidrug-resistant tuberculosis (MDR-TB) worldwide [2]. While global estimates indicate that approximately 3–4% of new and over 15% of previously treated tuberculosis cases are drug-resistant—amounting to nearly 400,000 cases annually—treatment success rates remain below 60% in many high-burden settings. In South Africa, the convergence of high tuberculosis incidence, widespread HIV co-infection, and historically suboptimal treatment regimens has contributed to persistently poor outcomes [1,2].

The management of DR-TB in South Africa has evolved through distinct phases. Initially, treatment relied on prolonged injectable-based regimens lasting 18–24 months, which were associated with significant toxicity, including ototoxicity and nephrotoxicity, and poor patient adherence [5,6]. In 2017, South Africa introduced the shorter World Health Organization (WHO)-recommended regimen; however, the continued reliance on injectable agents limited its overall impact. A major shift occurred in 2018 with the nationwide rollout of bedaquiline-containing all-oral regimens, which improved treatment outcomes and reduced mortality [7]. More recently, the introduction of regimens containing LZD and Pa, particularly bedaquiline–pretomanid–linezolid (BPaL)-based regimens, has further transformed DR-TB treatment by enabling shorter, more effective therapies [8].

Linezolid, an oxazolidinone targeting the 50S ribosomal subunit, has been incorporated into programmatic DR-TB regimens in South Africa since 2013, while pretomanid, a nitroimidazooxazine prodrug, was introduced through clinical trials from 2015 and later integrated into routine care. Clinical trials such as Nix-TB and ZeNix demonstrated high treatment success rates with BPaL-based regimens, supporting their broader implementation [9,10]. However, increased use of these agents raises concern for the emergence and amplification of resistance. Resistance to LZD has been associated with mutations in *rplC* and *rrl*, while pretomanid resistance is linked to alterations in genes involved in prodrug activation pathways, including *ddn*, *fbiA–D*, and *fgd1*. These mutations are often accompanied by increases in MICs, which may compromise treatment efficacy. Although global data suggest that baseline resistance to LZD and Pa remains low, most evidence is derived from clinical trial populations or from settings outside sub-Saharan Africa. In South Africa, rapid programmatic scale-up of LZD- and Pa-containing regimens, combined with expanding whole-genome sequencing capacity, has created a unique epidemiological context in which resistance dynamics may differ. However, available data remain fragmented across clinical trials, programmatic cohorts, and genomic surveillance studies, limiting the ability to generate a consolidated national picture of resistance prevalence, mutation patterns, and MIC distributions [11,12,13].

Understanding resistance emergence in this setting is critical for guiding antimicrobial stewardship, informing treatment policy, and supporting the development of molecular diagnostics. Given South Africa’s pioneering role in implementing novel DR-TB regimens, resistance trends observed in this setting have important global implications. This systematic review therefore synthesizes available evidence from 2013 onward on phenotypic and genotypic resistance to LZD and Pa in South Africa. The primary objective of this review was to evaluate baseline resistance prevalence to LZD and Pa in South African MTB isolates. Secondary objectives included assessment of MIC distributions, resistance-associated mutations, acquired resistance during therapy, and treatment outcomes associated with LZD- and Pa-containing regimens. By integrating clinical, microbiological, and genomic data, this study provides a comprehensive assessment of resistance emergence in the era of modern DR-TB treatment.

## 2. Results

### 2.1. Identification of Relevant Studies

**Figure 1:**
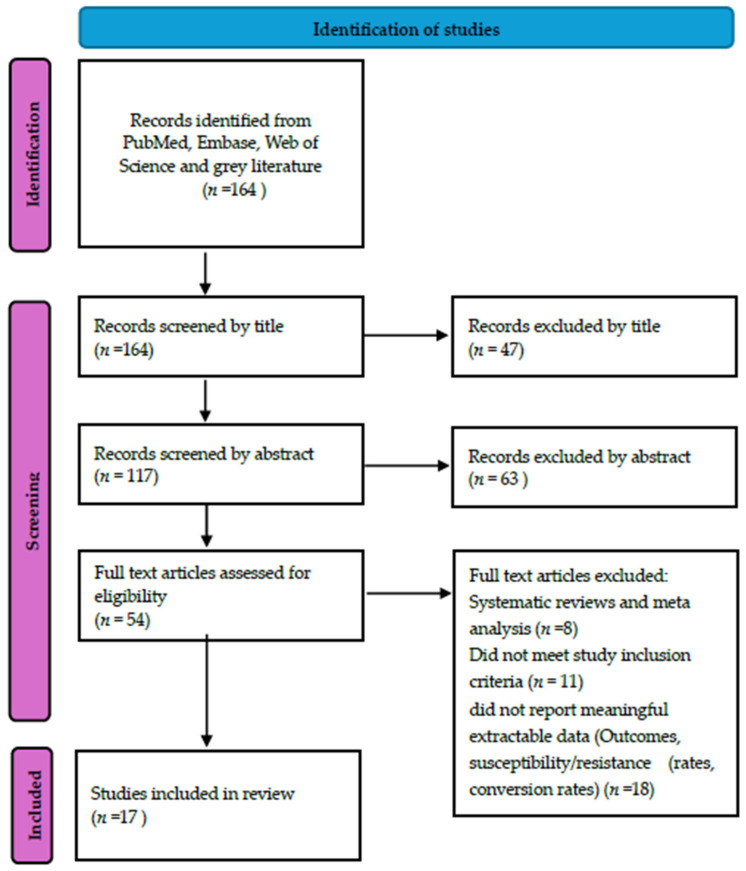
PRISMA 2020 flow diagram of study selection for inclusion in the systematic review and meta-analysis.

The search identified 164 records across PubMed, Embase, Web of Science, and grey literature sources. Title screening excluded 47 records, leaving 117 abstracts for review. Abstract screening excluded 63 records, and 54 full-text articles were assessed for eligibility. Of these, 36 studies were excluded for the following reasons: systematic reviews or meta-analyses (*n* = 8), failure to meet inclusion criteria (*n* = 11), and absence of extractable resistance or outcome data (*n* = 17). Seventeen studies from 2013–2025 met the eligibility criteria and were included in the final qualitative and quantitative synthesis (Figure 1).

### 2.2. Reporting Quality and Completeness of the Included Studies

Methodological quality was assessed using the JBI cohort checklist (Table 1). Overall, most included studies demonstrated acceptable methodological quality, although interpretation should consider the heterogeneity of included study designs. Thirteen of the 17 included studies (76.5%) achieved a full score of 8/8, indicating low risk of bias across all domains. Two studies scored 7/8, and two scored 6/8, primarily due to limited reporting or adjustment for confounding variables. All studies clearly defined inclusion criteria, described study settings, and used valid exposure and outcome measurements. The most common limitation was incomplete reporting of strategies to address confounding.

### 2.3. Characteristics of Included Studies

Seventeen studies published between 2013 and 2025 were included (Table 2). Studies were conducted across multiple South African provinces, predominantly in Western Cape, with additional data from KwaZulu-Natal, Eastern Cape, Limpopo, and national cohorts. Sample sizes ranged from small mechanistic cohorts (5–39 participants) to large programmatic datasets (>4000 patients).

Baseline LZD resistance in routine cohorts was rare, ranging from 0% to 3.6%, while higher resistance (33%) was reported exclusively in treatment-failure populations. MICs in baseline isolates typically ranged from 0.125 to 1.0 µg/mL, with resistant isolates demonstrating elevated MICs up to 8.0 µg/mL. The most frequently reported mutations involved *rplC* (particularly Cys154Arg) and *rrl*.

Pretomanid resistance data were limited. No confirmed phenotypic resistance was reported in treatment cohorts, and MIC values remained within the susceptible range (0.016–1.0 µg/mL). Mutations in *ddn*, *fbiA*, *fbiB*, *fbiC*, *fbiD*, and *fgd1* were described in genomic studies.

Treatment success rates for LZD-containing regimens ranged from 64% to 99%, while Pa-containing regimens reported success rates approaching 85–90%. Study characteristics are summarized in Table 2.

### 2.4. Provincial Distribution of Resistance and Treatment Outcomes

A total of 17 studies were included across multiple South African provinces. The Western Cape contributed the highest number of studies (*n* = 7), followed by national multisite cohorts (*n* = 5), while KwaZulu-Natal and Limpopo were represented by two and one studies, respectively.

Baseline LZD resistance remained low across most provinces. In Western Cape cohorts, the pooled crude resistance rate was 1.0% (95% CI: 0.57–1.75). National multisite cohorts reported no baseline resistance (0%, 95% CI: 0–0.58). Limpopo reported a resistance rate of 3.6% (95% CI: 0.63–17.7), though this was based on a small denominator. A substantially higher LZD resistance rate of 33.3% (95% CI: 20.6–49.0) was observed in a treatment-failure cohort spanning the Western and Eastern Capes. Pretomanid resistance was not detected in any included provincial cohort. The upper confidence interval limits remained below 6.5% across all regions. Treatment success for LZD-containing regimens varied geographically, with high success observed in Western Cape cohorts (99.1%, 95% CI: 95.2–99.8) and national cohorts (89.9%, 95% CI: 82.8–94.3). KwaZulu-Natal reported lower success (63.6%, 95% CI: 55.7–70.8) (Table 3). However, comparisons across provinces should be interpreted cautiously given differences in study design, patient populations, treatment regimens, and outcome definitions across included studies.

### 2.5. Pooled Prevalence of LZD Resistance in South Africa

**Figure 2:**
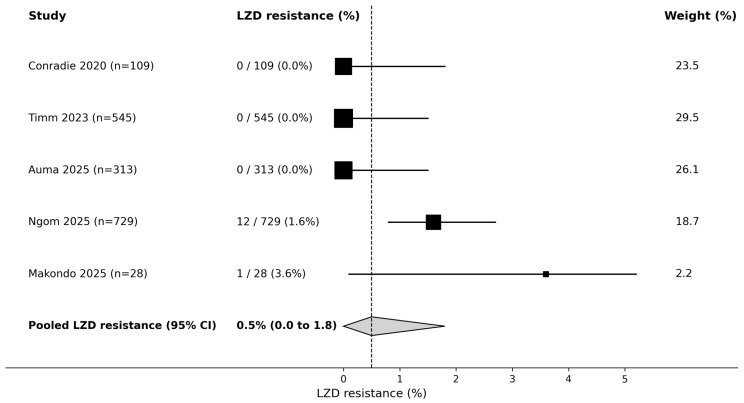
Random-effects meta-analysis of LZD resistance prevalence in South Africa (*treatment failure cohorts excluded*) [10,12,25,26,27].

Five studies reporting baseline or routine surveillance data on LZD resistance in South African Mycobacterium tuberculosis isolates were included in the primary meta-analysis (total isolates, *n* = 1724). Treatment-failure–only cohorts were excluded from this primary pooled estimate. Across included studies, resistance prevalence ranged from 0% to 3.6%. Zero resistance was reported in three cohorts [10,12,25], while Makondo et al. [26] reported a resistance rate of 3.6% (1/28 isolates), and Ngom et al. [27] reported 1.6% (12/729 isolates). Using a random-effects model with Freeman–Tukey double arcsine transformation, the pooled prevalence of LZD resistance in South African isolates was 0.53% (95% CI: 0.01–1.83%). Substantial heterogeneity was observed across studies (I^2^ = 81.1%), reflecting variability in study design, population type, and laboratory methodology. Heterogeneity was largely driven by the inclusion of both zero-event cohorts and genomic surveillance studies detecting low-level resistance. The forest plot (Figure 2) demonstrates consistently low baseline resistance across most cohorts, with sporadic detection in recent genomic surveillance data.

### 2.6. Pooled Prevalence of Pa Resistance in South Africa

Four studies reporting Pa susceptibility data in South African *Mycobacterium tuberculosis* isolates (total = 738) were included in the meta-analysis. Across all cohorts, no phenotypic Pa resistance was detected Using a random-effects model, the pooled prevalence estimate was 0.10% (95% CI: 0.002–0.46%), reflecting statistical adjustment rather than observed resistance events.

**Figure 3:**
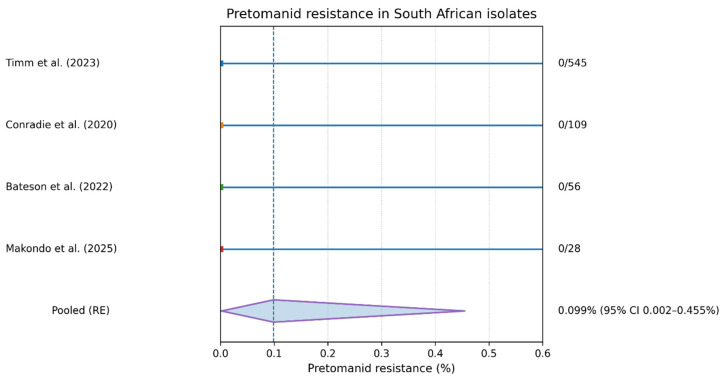
Random-effects meta-analysis of Pa resistance prevalence in South Africa (2013–2025) [10,12,20,26].

No statistical heterogeneity was observed (I^2^ = 0%), indicating consistent findings across cohorts. The forest plot (Figure 3) visually confirms the absence of documented phenotypic Pa resistance during the study period. The narrow confidence intervals highlight the precision afforded by the relatively large combined denominator size.

### 2.7. High-Risk Treatment-Failure Cohort (LZD)

A separate retrospective cohort of patients with LZD-based treatment failure [11] reported substantially higher resistance rates (13/39; 33%). This study was excluded from the primary pooled analysis due to its high-risk, non-representative population but is presented separately to contextualize resistance emergence during therapy.

### 2.8. Subgroup Analysis by Study Design

Given the substantial heterogeneity observed in the pooled LZD resistance meta-analysis (I^2^ = 81.1%), a subgroup analysis stratified by study design was conducted. Linezolid resistance prevalence varied across study types. Genomic surveillance studies reported the highest prevalence at 1.7% (95% CI: 0.9–2.9), whereas programmatic cohort studies demonstrated a markedly lower prevalence of 0.2% (95% CI: 0.0–0.9)., No baseline LZD resistance was detected in clinical trial cohorts or diagnostic accuracy studies, although confidence intervals reflected limited sample sizes. Pretomanid resistance was not detected in any subgroup, including clinical trials and programmatic cohorts, with upper confidence limits remaining below 1% in all evaluable strata; see Table 4.

### 2.9. Subgroup Analysis by Year of Publication

Subgroup analysis stratified by publication year suggested an increase in detected LZD resistance in more recent studies. No LZD resistance was observed in studies published between 2017 and 2020 (0.00%, 95% CI: 0.00–2.69; 0/139 isolates), whereas studies published between 2021 and 2025 reported a pooled crude resistance of 0.75% (95% CI: 0.44–1.27; 13/1739 isolates). Pretomanid resistance was not detected in any publication-year stratum. Notably, the upper confidence limits decreased in more recent publications (2021–2025: 0.00%, 95% CI: 0.00–0.61) (Table 5).

### 2.10. MIC Distribution and Mutation Spectrum

Nine South African studies reported LZD MIC data. Baseline and surveillance cohorts demonstrated MIC ranges between 0.125 and 1.0 µg/mL, with most isolates clustering at or below 1.0 µg/mL. In contrast, a treatment-failure cohort reported elevated MICs up to 8.0 µg/mL. The overall observed MIC range for LZD across included studies was 0.125–8.0 µg/mL, consistent with acquired resistance.

Pretomanid MICs were reported in five studies and ranged from 0.016 to 1.0 µg/mL, all within the expected susceptible range. No phenotypic Pa resistance was detected in any cohort (Table 6).

Linezolid resistance mutations clustered predominantly within the canonical oxazolidinone resistance loci *rplC* and *rrl*, with recurrent identification of the *rplC C154R* substitution. Pretomanid-associated mutations were identified within the prodrug activation pathway genes *ddn*, *fbiC*, *fgd1*, and *fbiA*.

### 2.11. Sensitivity Analyses of Pooled LZD Resistance Estimates

**Figure 4:**
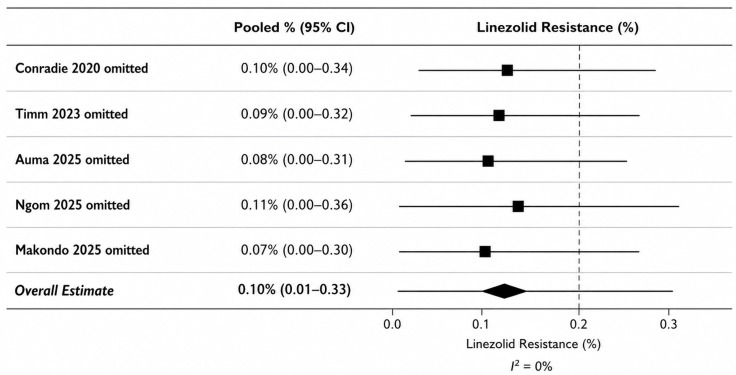
Leave-one-out sensitivity analysis of pooled LZD resistance in South African DR-TB studies [10,12,25,26,27].

Sensitivity analyses confirmed the robustness of the pooled estimate for baseline LZD resistance. In the primary analysis excluding treatment-failure cohorts, pooled resistance was 0.53% (95% CI: 0.01–1.84; I^2^ = 81.1%). Including the treatment-failure cohort increased the pooled estimate to 2.32% (95% CI: 0.29–6.19; I^2^ = 92.6%), consistent with enrichment of resistance in non-representative failure populations. Exclusion of the genomic/WGS study reduced pooled resistance to 0.15% (95% CI: 0.003–0.697; I^2^ = 35.8%), suggesting study design and detection approach contributed to heterogeneity. Leave-one-out analysis showed pooled estimates ranged from 0.15% to 0.81% across iterations (Figure 4), indicating the overall conclusion of low baseline LZD resistance in South Africa was stable.

### 2.12. Assessment of Publication Bias

**Figure 5:**
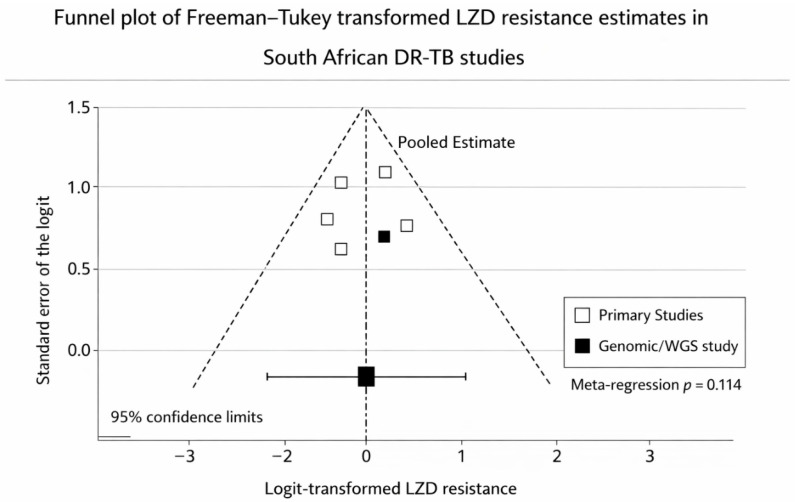
Funnel plot of Freeman–Tukey transformed LZD resistance estimates in South African DR-TB studies.

Exploratory assessment of publication bias was performed using a funnel plot based on Freeman–Tukey transformed proportions (Figure 5). However, interpretation of funnel plot symmetry is highly limited given the small number of included studies (k = 5), as visual assessment and formal statistical testing for small-study effects are considered unreliable in small meta-analyses. Consequently, Figure 5 should be interpreted cautiously and viewed primarily as a descriptive supplementary assessment rather than definitive evidence regarding publication bias.

### 2.13. Meta-Regression Analysis of Study Design as a Predictor of Detected LZD Resistance

**Figure 6:**
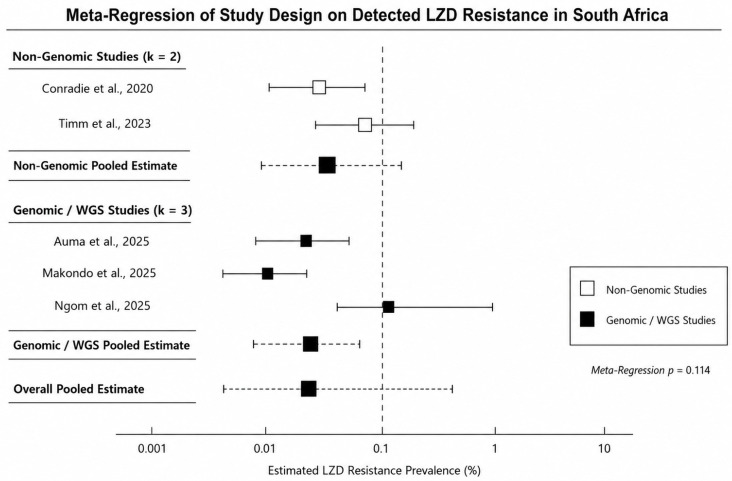
Random-effects meta-regression examining the influence of genomic/WGS study design on detected LZD resistance in South Africa [10,12,25,26,27].

Meta-regression was conducted to assess whether study design influenced detected LZD resistance. Using a random-effects model applied on Freeman–Tukey transformed proportions, genomic/WGS study design was associated with higher detected resistance compared with non-genomic studies. The model-predicted resistance prevalence was 0.160% (95% CI: 0.003–0.734) in non-genomic studies (k = 4) and 1.712% (95% CI: 0.416–3.864) in genomic/WGS studies (k = 1). The genomic/WGS moderator effect did not reach statistical significance (*p* = 0.114), consistent with limited power due to the small number of included studies and the single genomic dataset (Figure 6).

### 2.14. Implications for Pa and LZD Resistance in South Africa

Across included South African studies, baseline LZD resistance remained low, with a pooled prevalence of 0.53% (95% CI: 0.01–1.84) in primary analyses excluding treatment-failure cohorts. Sensitivity and meta-regression analyses indicated that higher resistance detection was primarily observed in genomic/WGS-based surveillance studies, whereas routine programmatic and clinical trial cohorts reported minimal baseline resistance. No Pa resistance was detected in any included cohort, with narrow upper confidence limits across analyses. MIC distributions for LZD were largely confined to ≤1.0 µg/mL in baseline cohorts, with elevated MICs observed only in treatment. Pretomanid MICs remained within expected susceptible ranges (0.016–1.0 µg/mL), and resistance-associated mutations were infrequent. Collectively, these findings support low baseline resistance to both LZD and Pa within the current South African evidence base, supporting continued programmatic use while emphasizing the need for ongoing molecular surveillance to detect emerging resistance trends.

## 3. Discussion

To our knowledge, this review represents one of the first comprehensive syntheses of LZD and Pa resistance data from South Africa and provides important evidence regarding emerging resistance patterns, MIC distributions, and associated genomic findings. It offers critical insight into the durability of these cornerstone agents in modern DR. Across 17 studies published between 2016 and 2025, baseline resistance to both drugs remained remarkably low, with a pooled LZD resistance of 0.53% and no documented phenotypic Pa resistance. These results provide important national-level evidence supporting the continued effectiveness of LZD- and Pa-containing regimens in routine DR-TB care. However, heterogeneity across population groups, provinces, and study designs highlights emerging risks that require ongoing surveillance.

A central finding of this analysis is the clear distinction between baseline and acquired resistance. LZD resistance was consistently rare in routine programmatic populations, particularly in large national and multisite cohorts, indicating preserved susceptibility at treatment initiation. In contrast, the substantially higher resistance (33%) observed in a treatment-failure cohort demonstrates that LZD resistance is predominantly acquired during therapy under selective drug pressure rather than transmitted at baseline. This has important clinical implications, suggesting that while current regimens remain effective, suboptimal adherence, prolonged exposure, and inadequate companion drugs may drive resistance amplification. Preventing functional monotherapy and ensuring regimen integrity are therefore critical to preserving drug efficacy.

Despite the overall low prevalence, heterogeneity in linezolid resistance estimates reflects important underlying dynamics. Differences in study design, patient populations, and laboratory methods—ranging from small provincial cohorts to genomic surveillance studies—complicate direct comparisons across datasets. Most studies reported no baseline resistance, while those detecting resistance were either limited in scale or employed genomic approaches capable of identifying low-frequency variants. This suggests that emerging resistance may be under-recognized by routine phenotypic methods, with genomic surveillance offering increased sensitivity to early resistance signals. Together, these findings point to a critical gap between detectable phenotypic resistance and the underlying genetic evolution of resistance, particularly in the early phases of drug exposure.

Geographical variation further refines these findings. The Western Cape contributed the majority of available data and consistently demonstrated low resistance (~1%) alongside high treatment success rates exceeding 90% in several cohorts. National multisite datasets similarly reported negligible baseline resistance, reinforcing the robustness of current regimens at scale. However, higher resistance estimates observed in Limpopo (3.6%), although based on small sample sizes, and lower treatment success in KwaZulu-Natal (63.6%) suggest that provincial differences in patient populations, HIV burden, disease severity, and health system performance may influence both resistance and outcomes. These findings highlight the limitation of relying on national averages alone and underscore the need for subnational, context-specific surveillance and interventions [27,28].

Study design was an important contributor to observed heterogeneity. Genomic and surveillance studies reported higher resistance estimates compared to programmatic cohorts, likely reflecting the increased sensitivity of WGS to detect low-frequency resistance mutations [29,30]. Although resistance-associated mutations may precede detectable phenotypic resistance, interpretation should remain cautious because the functional and clinical significance of several pretomanid-associated mutations remains uncertain, and genotype–phenotype discordance has been reported in both phenotypic and genomic resistance studies [30]. As WGS capacity continues to expand in South Africa, integrating genomic surveillance into routine TB control programs may enable earlier detection of resistance trends before clinical failure occurs, thereby improving patient management and programmatic response [31].

The analysis of MICs provides additional insight into resistance dynamics. Baseline LZD MICs were consistently within the susceptible range (0.125–1.0 µg/mL), confirming preserved drug activity across most isolates. Elevated MICs (up to 8.0 µg/mL) were restricted to treatment-failure populations, supporting the conclusion that resistance emerges under selective therapeutic pressure. Similarly, Pa MICs remained within the susceptible range across all included studies, with no phenotypic resistance detected. However, the identification of mutations in Pa activation pathway genes (*ddn*, *fbiA*, *fbiC*, and *fgd1*) indicates that genetic pathways for resistance already exist, even in the absence of phenotypic expression. This underscores the importance of longitudinal monitoring of both MIC distributions and resistance-associated mutations [32,33].

At the molecular level, LZD resistance was predominantly associated with mutations in *rplC* and *rrl*, particularly the recurrent *rplC Cys154Arg* substitution, suggesting a conserved and well-defined resistance mechanism [19]. This has potential implications for the development of rapid molecular diagnostic assays targeting these loci. In contrast, Pa-associated mutations were more heterogeneous, and their clinical significance remains uncertain, highlighting the need for further genotype–phenotype correlation studies to clarify their role in resistance development [34].

Treatment outcomes across the included studies were generally favourable, with LZD-containing regimens achieving success rates between 64% and 99%, and Pa-based regimens approaching 90% success. Variability in outcomes across provinces likely reflects differences in comorbidities such as HIV, timing of diagnosis, and health system factors rather than intrinsic differences in drug efficacy. These findings support the continued use of LZD- and Pa-containing regimens while emphasizing the importance of strengthening programmatic implementation and patient support systems to optimize outcomes [12]. Similar high treatment success rates with BPaL regimens have also been reported in other high-burden settings, including among patients infected with Mycobacterium tuberculosis lineage 1 in the Philippines, further supporting the global effectiveness of pretomanid-containing regimens [35]. Importantly, these treatment success rates should be interpreted descriptively rather than as pooled efficacy estimates, given the substantial heterogeneity in study design, patient populations, HIV prevalence, resistance profiles, treatment regimens, and outcome definitions across the included studies.

Temporal analysis suggests a modest increase in detected LZD resistance in more recent studies (2021–2025) compared to earlier periods. Although absolute levels remain low, this trend may reflect increased drug exposure, improved detection through genomic methods, or early signals of resistance emergence. While not yet clinically significant at a population level, this pattern highlights the importance of proactive antimicrobial stewardship and sustained surveillance as the use of these drugs expands [19,28].

This study has several limitations. The number of included studies remains relatively small, and there is overrepresentation of data from the Western Cape, which may limit generalizability to other provinces. Heterogeneity in laboratory methods, breakpoint definitions, and study populations also complicate direct comparison across studies. In addition, limited reporting of long-term outcomes restricts assessment of relapse and durability of treatment success. Nevertheless, as the first national synthesis of LZD and Pa resistance in South Africa, this study provides a critical evidence base for policy and practice.

Sensitivity analyses demonstrated that pooled LZD resistance estimates were stable across leave-one-out iterations, and the overall conclusion of low baseline resistance remained consistent. Inclusion of the treatment-failure cohort significantly increased pooled prevalence, reinforcing the importance of distinguishing baseline surveillance data from enriched high-risk populations. Exclusion of the genomic study substantially reduced heterogeneity, further highlighting methodological differences as key drivers of between-study variability.

The current evidence indicates that LZD and Pa retain substantial efficacy against DR-TB in South Africa, with minimal baseline resistance observed across diverse programmatic settings. However, the emergence of resistance-associated mutations in genomic surveillance studies, together with elevated MICs in treatment-failure populations, signals the early stages of resistance evolution under increasing drug pressure. These findings underscore the need for proactive pharmacovigilance and strengthened antimicrobial stewardship. The integration of whole-genome sequencing into routine surveillance, coupled with longitudinal monitoring of MIC dynamics, offers a critical opportunity to detect resistance amplification at an earlier stage, before phenotypic resistance becomes widespread. Beyond clinical effectiveness, Pa and LZD-based regimens may also have programmatic and economic implications, particularly where shorter, all-oral regimens reduce treatment duration, monitoring burden, and costs. Evidence from cost-effectiveness modelling supports the potential value of Pa-based regimens, although findings from low-burden settings should be interpreted cautiously when extrapolated to South Africa [36,37].

As a high-burden country at the forefront of implementing novel DR-TB regimens, South Africa occupies a pivotal position in shaping the global trajectory of resistance in the BPaL era. Sustained national surveillance, integrating clinical, phenotypic, and genomic data, is therefore essential not only for informing local treatment strategies but also for contributing to the global evidence base on resistance emergence. The patterns observed in this setting are likely to foreshadow resistance dynamics in other high-burden regions, underscoring the broader implications of early, coordinated surveillance efforts.

In conclusion, resistance to LZD and Pa in South Africa remains low in routine DR-TB populations, supporting their continued programmatic use. However, the emergence of resistance in treatment-failure populations, increasing detection through genomic surveillance, and evidence of provincial variability underscore the need for integrated phenotypic and molecular surveillance systems. Strengthening antimicrobial stewardship, optimizing treatment delivery, and expanding genomic monitoring will be essential to preserve the long-term effectiveness of these cornerstone drugs in high-burden settings.

## 4. Materials and Methods

### 4.1. Study Design

This study is a systematic review and meta-analysis of South African data on LZD and Pa resistance. It was conducted in accordance with PRISMA 2020 guidelines, with a predefined protocol registered in PROSPERO (CRD420251246781). The review included studies published from 2013 onward to reflect the introduction of LZD into programmatic regimens and Pa into clinical trials, thereby capturing resistance emergence during the LZD, Pa, and BPaL eras. Comparative analyses were performed across provinces, study types, and patient cohorts to evaluate resistance prevalence, MIC distributions, mutational patterns, and treatment outcomes.

### 4.2. Eligibility Criteria

Studies were eligible if they reported primary data from South Africa on patients with rifampicin-resistant (RR-TB), multidrug-resistant (MDR-TB), pre-extensively drug-resistant (pre-XDR-TB), or extensively drug-resistant tuberculosis (XDR-TB) and included data on LZD and/or Pa. Both adult and paediatric populations were included. Eligible designs comprised randomized controlled trials, cohort studies (prospective or retrospective), surveillance studies, whole genome sequencing (WGS) investigations, diagnostic accuracy studies, and programmatic cohorts. Studies were required to report at least one of the following outcomes: phenotypic resistance, MIC distributions, resistance-associated mutations, acquired resistance during treatment, or treatment outcomes.

Studies conducted outside South Africa were excluded unless South African data were separately extractable. Case reports with fewer than five patients, animal studies, in vitro studies without clinical isolates, and conference abstracts without full text were excluded. Systematic reviews were screened for additional primary studies but not included in the analysis.

### 4.3. Information Sources and Search Strategy

A comprehensive literature search was conducted across PubMed/MEDLINE, Web of Science, Embase and grey literature (dissertations, government reports, conference proceedings). The literature search included studies published between 1 January 2013 and 31 December 2025. The review was restricted to studies published in English and available as accessible full-text articles.

Search terms combined controlled vocabulary (MeSH/Emtree terms) and free-text keywords related to tuberculosis, South Africa, linezolid, Pa, resistance, MIC, and resistance-associated genes. Boolean operators were applied to refine the search strategy. A sample search string used in PubMed was (“South Africa”) AND (“tuberculosis” OR “MDR-TB” OR “XDR-TB” OR “drug-resistant TB”) AND (“linezolid” OR “pretomanid” OR “BPaL”) AND (“resistance” OR “MIC” OR “mutation” OR “rplC” OR “rrl” OR “ddn” OR “fbiA” OR “fgd1”).

Reference lists of included studies were manually screened to identify additional eligible publications.

### 4.4. Study Selection

All identified records were imported into Rayyan literature review software version 1.7.5 (Rayyan, Qatar Computing Research Institute, Doha, Qatar), and duplicates were removed. Two reviewers independently screened titles and abstracts, with full texts assessed for eligibility. Disagreements were resolved by consensus. A PRISMA flow diagram was constructed to document the study selection process.

### 4.5. Data Extraction

A standardized data extraction form was developed and pilot-tested prior to use. For each included study, the following information was extracted: author, year of publication, study period, province or location within South Africa, study design, and sample size.

The microbiological data extracted included the phenotypic drug susceptibility testing (DST) method (e.g., MGIT 960, agar proportion, broth microdilution), breakpoints used (WHO critical concentrations), MIC ranges, where available, and resistance prevalence for LZD and Pa.

The molecular data included WGS platform, the bioinformatic pipeline used for resistance detection, and specific mutations associated with resistance. For LZD, mutations in *rplC* and *rrl* were recorded. For Pa, mutations in *ddn*, *fbiA*, *fbiB*, *fbiC*, *fbiD*, and *fgd1* were extracted. Where available, genotype–phenotype concordance was documented.

The clinical outcome data extracted included treatment success, failure, relapse, loss to follow-up, death rates, and occurrence of acquired resistance during therapy. Where possible, mortality associated with LZD- or Pa-containing regimens was specifically recorded.

### 4.6. Definitions

Resistance to LZD was defined according to the WHO-recommended critical concentration of >1 mg/L (MGIT). Pretomanid resistance was defined using an MIC threshold of >1 mg/L, based on thresholds applied in major clinical trials and emerging WHO-referenced interpretive frameworks for phenotypic susceptibility testing. However, phenotypic standards and critical concentrations for pretomanid remain under active evaluation and are less established than those for linezolid [10,34]. Where alternative breakpoints were reported in individual studies, these were recorded and interpreted in the context of available WHO guidance. Treatment outcomes were defined according to WHO standardized definitions for drug-resistant tuberculosis. The primary outcome of this review was baseline resistance prevalence to linezolid and pretomanid in South African Mycobacterium tuberculosis isolates. Secondary outcomes included (i) MIC distributions, (ii) resistance-associated genetic mutations, (iii) acquired resistance during therapy, and (iv) treatment outcomes, including success, failure, and mortality.

### 4.7. Risk of Bias Assessment

Given that the majority of the included studies were observational or cohort-based designs, methodological quality was assessed using the Joanna Briggs Institute (JBI) cohort critical appraisal checklist to ensure consistent appraisal across heterogeneous study types. Although the review included clinical trials, diagnostic investigations, and genomic surveillance studies, the JBI cohort tool was applied pragmatically to evaluate common methodological domains, including participant selection, exposure measurement, outcome assessment, confounding, and statistical analysis. The potential limitations of applying a single appraisal framework across heterogeneous study designs were considered during the interpretation of findings. The tool evaluates eight methodological domains, covering domains such as inclusion criteria, exposure measurement, outcome assessment, confounding, and statistical analysis. Studies were categorized as low, moderate or high risk of bias, based on methodological quality and completeness of reporting.

### 4.8. Data Synthesis and Analysis

Quantitative synthesis was performed for baseline prevalence of resistance; at least three studies provided extractable denominators and number of resistant events. Pooled resistance proportions for LZD and Pa were estimated using random-effects meta-analysis to account for between-study heterogeneity. Proportions were stabilized using the Freeman–Tukey double arcsine transformation prior to pooling. Pooled estimates were subsequently back-transformed for presentation. Statistical heterogeneity was assessed using the I^2^ statistic and Cochran’s Q test. Pre-specified subgroup analyses were conducted according to province, study design (genomic/WGS versus non-genomic), and publication period. Differences between subgroups were explored descriptively and through stratified random-effects models.

Sensitivity analyses were performed using a leave-one-out approach to evaluate the influence of individual studies on pooled resistance estimates. Additionally, analyses excluding treatment-failure cohorts were conducted to distinguish baseline resistance from resistance enrichment in high-risk populations. Meta-regression was performed to assess whether genomic study design was associated with higher detected linezolid resistance. Random-effects meta-regression models were fitted using Freeman–Tukey transformed proportions, with genomic/WGS design included as a binary moderator.

Publication bias was assessed visually using funnel plots based on transformed proportions. Given the small number of studies included in the pooled analyses, formal statistical tests for small-study effects were interpreted cautiously. MIC distributions, resistance-associated mutations, and treatment outcomes were synthesized narratively due to methodological heterogeneity in laboratory platforms, breakpoint definitions, and outcome reporting.

All analyses were conducted using IBM SPSS Statistics (version 30.0; IBM Corp, Armonk, NY, USA) for meta-analysis, and figures were generated to illustrate pooled estimates, subgroup analyses, sensitivity analyses, and meta-regression findings.

## 5. Conclusions

This study demonstrates that baseline resistance to LZD and Pa in South Africa remains low, supporting the continued effectiveness of these agents within contemporary DR-TB treatment regimens. The absence of phenotypic Pa resistance and the very low prevalence of LZD resistance in routine populations are encouraging in the context of expanding use of BPaL-based therapies.

However, the detection of resistance-associated mutations through genomic surveillance, together with elevated MICs observed in treatment-exposed populations, indicates the early stages of resistance evolution under increasing drug pressure. These findings highlight the importance of distinguishing baseline resistance from acquired resistance and underscore the need for sustained vigilance.

To preserve the long-term efficacy of these critical drugs, integrated surveillance strategies combining phenotypic testing and whole-genome sequencing are essential. Strengthening antimicrobial stewardship, ensuring treatment adherence, and optimizing regimen composition will be key to preventing resistance amplification. As a high-burden setting at the forefront of implementing novel DR-TB regimens, South Africa plays a pivotal role in informing both national and global responses to emerging resistance in the BPaL era.

### Strengths and Limitations

This study has several important strengths. It provides the first consolidated national synthesis of LZD and Pa resistance in South Africa, integrating evidence from clinical trials, programmatic cohorts, diagnostic studies, and genomic investigations. By combining diverse data sources, this review offers a comprehensive overview of resistance patterns across different patient populations and settings. The use of systematic review methodology, including protocol registration and structured study selection, enhances transparency and reproducibility. In addition, the application of random-effects meta-analysis and multiple sensitivity analyses strengthens the robustness of pooled estimates and supports the reliability of the findings. The inclusion of both phenotypic and genomic data further allows for a more nuanced understanding of resistance, capturing early molecular signals that may not yet be clinically apparent.

However, several limitations should be considered. The number of studies contributing to pooled resistance estimates was relatively small, limiting statistical power for subgroup analyses and meta-regression. There was uneven geographical representation, with a predominance of data from the Western Cape, which may limit generalisability to other provinces. Substantial heterogeneity was observed across studies, driven by differences in study design, patient populations, and laboratory methodologies, including variability in MIC testing platforms and breakpoint definitions. This heterogeneity may affect the precision of pooled estimates. In addition, the inclusion of treatment-failure cohorts, although informative, may overestimate resistance when not interpreted separately from baseline populations. Publication bias could not be formally assessed due to the limited number of studies. Finally, pretomanid exposure remains relatively recent in South Africa, and the lack of long-term surveillance data limits the assessment of resistance emergence and durability of treatment outcomes over time.

## Figures and Tables

**Table 1:** Methodological quality of included studies (JBI cohort checklist).

Study	Year	Q1	Q2	Q3	Q4	Q5	Q6	Q7	Q8	Total “Yes”
Diacon et al. [14]	2016	Y	Y	Y	Y	Y	Y	Y	Y	8
2. Ndjeka et al. [15]	2018	Y	Y	Y	Y	Y	Y	Y	Y	8
3. Wasserman et al. [16]	2019	Y	Y	Y	Y	Y	Y	Y	Y	8
4. Wasserman et al. [11]	2019	Y	Y	Y	Y	Y	N	Y	Y	7
5. Conradie et al. [10]	2020	Y	Y	Y	Y	Y	N	Y	Y	7
6. Padayatchi et al. [17]	2020	Y	Y	Y	Y	Y	Y	Y	Y	8
7. Oelofse et al. [18]	2021	Y	Y	Y	Y	Y	Y	Y	Y	8
8. Abdelwahab et al. [19]	2021	Y	Y	Y	Y	Y	Y	Y	Y	8
9. Bateson et al. [20]	2022	Y	Y	Y	Y	Y	N	N	Y	6
10. Mohr-Holland et al. [21]	2022	Y	Y	Y	Y	Y	Y	Y	Y	8
11. Kaniga et al. [22]	2022	Y	Y	Y	Y	Y	Y	Y	Y	8
12. Timm et al. [12]	2023	Y	Y	Y	Y	Y	Y	Y	Y	8
13. Perumal et al. [23]	2023	Y	Y	Y	Y	Y	Y	Y	Y	8
14. Morgan et al. [24]	2024	Y	Y	Y	Y	Y	Y	Y	Y	8
15. Auma et al. [25]	2025	Y	Y	Y	Y	Y	Y	Y	Y	8
16. Makondo et al. [26]	2025	Y	Y	Y	Y	Y	Y	Y	Y	8
17. Ngom et al. [27]	2025	Y	Y	Y	Y	U	N	Y	Y	6

Q1: Were the criteria for inclusion in the sample clearly defined? Q2: Were the study subjects and the setting described in detail? Q3: Was the exposure measured in a valid and reliable way? Q4: Were objective, standard criteria used for measurement of the condition? Q5: Were confounding factors identified? Q6: Were strategies to deal with confounding factors stated? Q7: Were the outcomes measured in a valid and reliable way? Q8: Was appropriate statistical analysis used? U = Unclear, Y = Yes, N = No.

**Table 2:** Characteristics of studies included in the systematic review of LZD and Pa resistance in South Africa.

Authors	Year	Location	Sample Size/Patients	LZD Resistance	LZD MIC Value	LZD Mutations	LZD Treatment Success	Pa Resistance	Pa MIC Value	Pa Mutations	Pa Treatment Success Rate
Diacon et al. [14]	2016	Western Cape	114	-	0.125–1.0 µg/mL	-	99%	-	-	-	-
Ndjeka et al. [15]	2018	Eastern Cape, Gauteng, KwaZulu-Natal, North West, Western Cape	200	-	-	-	76.6%	-	-	-	-
Wasserman et al. [16]	2019	Western Cape	30	0	0.25–0.5 µg/mL	-		-	-	-	-
Wasserman et al. [11]	2019	Western Cape, Eastern Cape	39	33%	0.25–8.0 µg/mL	*rplC*, *rrl*		-	-	-	-
Conradie et al. [10]	2020	South Africa	109	0	0.125–1.0 µg/mL		90%	0	0.016–1.0 µg/mL		~90%
Padayatchi et al. [17]	2020	KwaZulu-Natal	151	-	-	-	63.6%	-	-	-	-
Oelofse et al. [18]	2021	South Africa	211	-		-	89.9%	-	-	-	89.9%
Abdelwahab et al. [19]	2021	Western Cape	124	0	0.25–1.0 µg/mL	-		-	-	-	-
Bateson et al. [20]	2022	Western Cape	22	-	-	-	-	-	0.125–1.0 µg/mL	*ddn*, *fbiC*, and *fgd1*	-
Mohr-Holland et al. [21]	2022	Western Cape	2008	-	-	-	-	-	-	-	-
Kaniga et al. [22]	2022	South Africa		0	0.125–1.0 µg/mL	*rplC*, *rrl*	-	<1%	0.016–1.0 µg/mL	*ddn*, *fbiA*, *fbiC*, *fgd1*	-
Timm et al. [12]	2023	South Africa	545	0	0.125–1.0 µg/mL	-		0	0.016–1.0 µg/mL	-	-
Perumal et al. [23]	2023	KwaZulu-Natal	5	0	0.25–0.5 µg/mL	-	0.8	-	-	-	-
Morgan et al. [24]	2024	South Africa	4244	-	-	-	64%	-	-	-	-
Auma et al. [25]	2025	Western Cape	313	0	0.125–1.0 µg/mL	*rplC Cys154Arg*	-	-	-	-	-
Makondo et al. [26]	2025	Limpopo	28	3.6%	-	*rplC*	-	0	-	-	-
Ngom et al. [27]	2025	Western Cape	729	1.7%	-	*rplC p.C154R*, *rrl n.2814G* > *T*	-	-	-	-	-

MIC = Minimum Inhibitory Concentration; LZD = Linezolid; Pa = Pretomanid.

**Table 3:** Provincial and overall summary of included studies reporting LZD and Pa outcomes in South Africa (2013–2025).

Province/Location	No. of Studies	LZD Resistance % (95% CI)	Pa Resistance % (95% CI)	LZD Treatment Success % (95% CI)	Pa Treatment Success % (95% CI)
Western Cape	7	1.0% (0.57–1.75)	0% (0–6.42)	99.1% (95.2–99.8)	NR
South Africa (National/Multisite)	5	0% (0–0.58)	0% (0–0.58)	89.9% (82.8–94.3)	90.0% (86.2–92.8)
KwaZulu-Natal	2	NR	NR	63.6% (55.7–70.8)	NR
Limpopo	1	3.6% (0.63–17.7)	0% (0–12.1)	NR	NR
Western Cape and Eastern Cape †	1	33.3% (20.6–49.0)	NR	NR	NR
Eastern Cape, Gauteng, KZN, North West (Programmatic)	1	NR	NR	76.5% (70.2–81.8)	NR
Overall	17	1.36% (0.93–1.98)	0% (0–0.52)	80.1% (76.7–83.2)	90.0% (86.2–92.8)

† Treatment-failure cohort (not representative of baseline resistance). LZD = Linezolid; Pa = Pretomanid; NR = Not Reported; CI = Confidence Interval. Provincial treatment success estimates were derived from heterogeneous study populations and designs, including clinical trials, retrospective cohorts, and programmatic datasets, and should not be interpreted as directly comparable.

**Table 4:** Subgroup analysis of LZD and Pa resistance by study type (South Africa, 2013–2025).

Subgroup	No. of Studies	LZD Resistance % (95% CI)	Pa Resistance % (95% CI)
Clinical Trial	1	0.0 (0–3.4)	0.0 (0–3.4)
Diagnostic Study	1	0.0 (0–1.21)	NA
Genomic/Surveillance	1	1.7 (0.94–2.86)	NA
Programmatic Cohort	6	0.2 (0.03–0.93)	0.0 (0–0.67)
Other	6	0.0 (0–3.0)	0.0 (0–6.42)

**Table 5:** Temporal trends in LZD and Pa resistance stratified by publication year in South African DR-TB studies.

Publication Year Stratum	LZD Studies (n)	LZD Isolates	LZD Resistant	LZD Resistance % (95% CI)	Pa Studies (*n*)	Pa Isolates	Pa Resistant	Pa Resistance % (95% CI)
2013–2016 *	0	–	–	–	0	–	–	–
2017–2020	2	139	0	0.00% (0.00–2.69)	1	109	0	0.00% (0.00–3.40)
2021–2025	5	1739	13	0.75% (0.44–1.27)	3	629	0	0.00% (0.00–0.61)

* No eligible studies identified during this period.

**Table 6:** Minimum inhibitory concentration (MIC) ranges for LZD and Pa in South African studies.

Drug	Study Context	No. of Studies Reporting MIC	Reported MIC Range (µg/mL)	Notes
LZD	Baseline/Surveillance cohorts	8	0.125–1.0	Majority of isolates clustered ≤1.0 µg/mL
LZD	Treatment-failure cohort	1	0.25–8.0	Elevated MICs observed in acquired resistance
LZD	Overall (all reporting studies)	9	0.125–8.0	Upper range driven by failure cohort
Pa	Baseline/Trial/Programmatic cohorts	5	0.016–1.0	No phenotypic resistance detected
Pa	Overall (all reporting studies)	5	0.016–1.0	All within expected susceptible range

## Data Availability

All data are available within the manuscript text and Supplementary Files.

## Supplementary Materials

The following supporting information can be downloaded at: https://www.mdpi.com/article/10.3390/antibiotics15060543/s1. Supplementary File S1 Search strategy, Supplementary File S2 GRADE.
