# Supplementary material for: Resistance to Linezolid and Pretomanid in the Era of Modern Drug-Resistant Tuberculosis Treatment in South Africa: A Systematic Review and Meta-Analysis
Source: Antibiotics (Basel). 2026 May 28;15(6):543. doi: 10.3390/antibiotics15060543 (PMC13296143; doi:10.3390/antibiotics15060543)
Supplement: Supplementary file 1 [file antibiotics-15-00543-s001.zip › Supplementary file S1 Search strategy.pdf]

Supplementary File S2: Detailed Search Strategy

Search Strategy for Systematic Review of Linezolid and Pretomanid Resistance in South Africa (2013–2025)

Pubmed

("South Africa"[MeSH] OR "South Africa"[tiab])

AND

("Tuberculosis"[MeSH] OR tuberculosis[tiab] OR "MDR-TB"[tiab] OR "XDR-TB"[tiab] OR "drug-resistant tuberculosis"[tiab])

AND

("Linezolid"[MeSH] OR linezolid[tiab]

OR "Pretomanid"[Supplementary Concept] OR pretomanid[tiab]

OR "BPaL"[tiab] OR "bedaquiline pretomanid linezolid"[tiab])

AND

("Drug Resistance, Microbial"[MeSH] OR resistance[tiab] OR "drug resistance"[tiab]

OR MIC[tiab] OR "minimum inhibitory concentration"[tiab]

OR mutation*[tiab] OR rplC[tiab] OR rrl[tiab]

OR ddn[tiab] OR fbiA[tiab] OR fbiC[tiab] OR fgd1[tiab])

EMBASE

('south africa'/exp OR 'south africa':ti,ab)

AND

('tuberculosis'/exp OR tuberculosis:ti,ab OR 'multidrug resistant tuberculosis':ti,ab OR 'extensively drug resistant tuberculosis':ti,ab)

AND

('linezolid'/exp OR linezolid:ti,ab

OR 'pretomanid'/exp OR pretomanid:ti,ab

OR 'bpal':ti,ab OR 'bedaquiline pretomanid linezolid':ti,ab)

AND

('drug resistance'/exp OR resistance:ti,ab

OR 'minimum inhibitory concentration'/exp OR mic:ti,ab

OR mutation*:ti,ab OR rplc:ti,ab OR rrl:ti,ab

OR ddn:ti,ab OR fbia:ti,ab OR fbic:ti,ab OR fgd1:ti,ab)

Web of Science

TS=("South Africa")

AND TS=("tuberculosis" OR "MDR-TB" OR "XDR-TB" OR "drug-resistant TB")

AND TS=("linezolid" OR "pretomanid" OR "BPaL" OR "bedaquiline pretomanid linezolid")

AND TS=("resistance" OR "MIC" OR "minimum inhibitory concentration" OR "mutation" OR rplC OR rrl OR ddn OR fbiA OR fbiC OR fgd1)

Grey literature

"South Africa" AND tuberculosis AND (linezolid OR pretomanid OR BPaL) AND resistance
