# Supplementary material for: Resistance to Linezolid and Pretomanid in the Era of Modern Drug-Resistant Tuberculosis Treatment in South Africa: A Systematic Review and Meta-Analysis
Source: Antibiotics (Basel). 2026 May 28;15(6):543. doi: 10.3390/antibiotics15060543 (PMC13296143; doi:10.3390/antibiotics15060543)
Supplement: Supplementary file 1 [file antibiotics-15-00543-s001.zip › Supplementary file S2 GRADE.pdf]

Supplementary Table S1: GRADE Summary of Evidence for Linezolid and Pretomanid Resistance in South Africa (2013–2025)

Outcome	Study Design	Risk of Bias	Inconsistency	Indirectness	Imprecision	Publication Bias	Effect Estimate	Certainty of Evidence (GRADE)
**Baseline LZD resistance**	Observational (cohort, surveillance, genomic)	Not serious	**Serious** (I^2 = 81.1%)	Not serious	**Serious** (wide CI, rare events)	Uncertain	0.53% (95% CI: 0.01–1.83)	**LOW**
**Baseline Pa resistance**	Observational + trials	Not serious	Not serious (I^2 = 0%)	Not serious	**Serious** (no events)	Uncertain	0.10% (95% CI: 0.002–0.46)	**MODERATE**
**LZD MIC distribution**	Observational laboratory studies	Not serious	Not serious	Not serious	Not serious	Uncertain	0.125–1.0 µg/mL (baseline); up to 8.0 µg/mL (failure)	**MODERATE**
**Pa MIC distribution**	Observational + trials	Not serious	Not serious	Not serious	**Serious** (limited data)	Uncertain	0.016–1.0 µg/mL (no resistance detected)	**MODERATE**
**Resistance mutations (genotypic)**	Genomic/ WGS studies	Not serious	**Serious** (variable detection)	Not serious	**Serious** (limited correlation data)	Uncertain	LZD: *rplC, rrl*; Pa: *ddn, fbiA–D, fgd1*	**LOW**
**Treatment outcomes**	Cohort + trials	Not serious	**Serious** (provincial	Not serious	Not serious	Uncertain	LZD: 64–99%; Pa:	**MODERATE**

			variation<br>)				~90%<br>succes<br>s	
